# Prognostic impact of progesterone receptor status in patients with breast cancer and isolated locoregional recurrence

**DOI:** 10.1007/s12282-026-01882-z

**Published:** 2026-06-21

**Authors:** Takeshi Murata, Hiraku Kumamaru, Masayuki Yoshida, Naoko Kinukawa, Shin Takayama, Akihiko Suto, Hiromitsu Jinno, Takanori Ishida, Naoki Niikura, Shigehira Saji

**Affiliations:** 1 Department of Breast Surgery, National Cancer Center Hospital, 5-1-1, Tsukiji, Chuo-Ku, Tokyo, Japan; 2 Department of Health Data Science, Yokohama City University Graduate School of Data Science, 22-2, Seto, Kanazawa-Ku, Yokohama, Kanagawa Japan; 3 Department of Healthcare Quality Assessment, The University of Tokyo Graduate School of Medicine, 7-3-1, Hongo, Bunkyo-Ku, Tokyo, Japan; 4 Department of Diagnostic Pathology, National Cancer Center Hospital, 5-1-1, Tsukiji, Chuo-Ku, Tokyo, Japan; 5 Department of Surgery, Teikyo University School of Medicine, 2-11-1 Kaga, Itabashi-Ku, Tokyo, Japan; 6 Department of Breast and Endocrine Surgical Oncology, Tohoku University Graduate School of Medicine, Sendai, Japan; 7 Department of Breast Oncology, Tokai University School of Medicine, 143, Shimokasuya, Isehara, Kanagawa Japan; 8 Department of Medical Oncology, School of Medicine, Fukushima Medical University, Fukushima, Japan

**Keywords:** Breast cancer, Isolated locoregional recurrence, Progesterone receptor, Overall survival, Breast cancer-specific survival

## Abstract

**Background:**

The impact of progesterone receptor (PR) status on the prognosis of breast cancer after isolated locoregional recurrence (ILRR) remains unclear. This study assessed the prognostic impact of the PR status of ILRR tumors in patients with estrogen receptor-positive (ER+)/human epidermal growth factor receptor 2-negative (HER2-) breast cancer.

**Methods:**

The study utilized data from the Japanese National Clinical Database (2004–2015), including 1,625 patients with ER+/HER2- ILRR. All participants were patients aged between 20 and 75 years at diagnosis who underwent curative surgery for ER+/HER2- primary breast cancer. Patients were classified into three groups based on the PR status of primary (p) and recurrent (r) tumors. We compared the overall survival (OS) and breast cancer-specific survival (BCSS) among the three groups using the Kaplan–Meier method with the log-rank test. ER and PR were considered positive if immunohistochemistry staining was positive in > 1% of tumor cells. For the ER expression levels in ILRR tumors, 1–10% were considered as ER-low positive.

**Results:**

Patients with PR- ILRR (pPR-/rPR- and pPR+/rPR-) had significantly worse OS and BCSS than those with PR+ ILRR (pPR+/rPR+). Factors associated with worse outcomes included lymph node metastasis, a shorter disease-free interval, ER-low positivity, and no surgery for ILRR tumor.

**Conclusions:**

This study highlights PR-negativity in ER+/HER2- ILRR tumors as a poor prognostic factor after ILRR, independent of the PR status of the primary breast cancer, emphasizing the need for tailored treatment strategies.

**Supplementary Information:**

The online version contains supplementary material available at https://doi.org/10.1007/s12282-026-01882-z.

## Introduction

The reported incidence of locoregional recurrence (LRR) in breast cancer ranges from 3% to 10% [1–3]. Isolated LRR (ILRR) is associated with a higher risk of distant recurrence and mortality [3–6]. Furthermore, overall survival (OS) after ILRR is reported to be relatively low [7]. However, clinical evidence on treatment strategies for ILRR is lacking owing to the varying recurrence patterns and treatments for each patient.

Prospective trials, including the SAKK 23/82 [8] and CALOR trials [9], have evaluated systemic therapy for ILRR. The SAKK 23/82 trial showed that tamoxifen (TAM) administration significantly improved 5-year relapse-free survival (RFS) in estrogen receptor-positive (ER+) ILRR after resection of recurrent tumors, compared with no TAM (61% vs. 40%) [8]. The CALOR trial showed a significant improvement in 10-year RFS for ER- ILRR with complete resection of the ILRR tumor followed by irradiation and chemotherapy (70% vs. 34%), while no significant improvement was observed in ER+ ILRR (50% vs. 59%) [9].

However, the CALOR trial has several limitations [9], including a small sample size (only 17% of the expected number of patients), a 12% loss to follow-up among patients with ER+ ILRR, and failure to evaluate the effect of chemotherapy in Luminal B-like breast cancer (ER + and progesterone receptor-negative (PR-) breast cancer). Although previous reports have indicated that PR-negativity in primary breast cancer and PR loss in metastatic sites are poor prognostic factors [10, 11], reports on the significance of PR status in ER+ ILRR tumors have been limited to studies with small cohorts [12, 13]. In the CALOR trial, the second ILRR rate and distant recurrence rate for ER+/PR+ (*n* = 73) and ER+/PR- ILRR tumors (*n* = 28) were both higher in the latter group (0% vs. 21%, 15% vs. 29%, respectively) [12]. In another retrospective study (*n* = 306), PR- ILRR tumors were reported as a risk factor associated with poor distant metastasis-free survival [13].

The aim of this study was to test, in a large cohort, the following hypothesis: Among patients with ER+/human epidermal growth factor receptor 2-negative (HER2-) ILRR tumors, those who were PR + in both the primary and ILRR tumors would have a better prognosis than (1) patients who were PR- in both tumors and (2) patients who were PR + in the primary tumor but became PR- in the ILRR tumors.

## Patients and methods

### Study design and patients

This study was approved by the National Cancer Center Hospital Review Board and Ethical Committee (approval no. 2021-014). The need for informed consent was waived owing to the anonymized nature of the patient database. The participants were patients aged between 20 and 75 years at diagnosis who underwent curative surgery for ER+/HER2- primary breast cancer and were registered in the Japanese Breast Cancer Registry between January 1, 2004 and December 31, 2015. Patients with bilateral breast cancer (both synchronous and metachronous), ductal carcinoma in situ, lobular carcinoma in situ, stage IV disease, inflammatory breast cancer, male breast cancer, no axillary surgery at primary surgery, no recurrence, unknown primary PR status, or a recurrence site other than ILRR were excluded. Patients without ILRR tumor biopsy or with ER-negative or HER2-positive ILRR tumors were also excluded. Additionally, patients whose primary PR status was negative but whose recurrent PR status was positive were excluded, as such cases are more consistent with a new primary tumor (NP) than with a true recurrence (TR) [14–21]. ER and PR were considered positive if immunohistochemistry (IHC) staining was positive in > 1% of tumor cells [22]. Furthermore, for the ER expression levels in ILRR tumors, 1–10% were considered as ER-low positive. A HER2- result corresponded to a score of 0 or 1 + on IHC or 2 + on IHC without amplification on fluorescence in situ hybridization [23, 24]. Prior to the publication of the guidelines, decisions were made based on the criteria for in vitro diagnostic reagents. All pathological assessments, including ER, PR, and HER2 status, were performed at each participating institution according to the Japanese guidelines for breast cancer classification and reporting, which are aligned with internationally accepted standards such as the ASCO/CAP and UICC criteria. Because this study utilized registry-based data, no centralized pathological review was conducted. The tumor, node, and metastasis staging of breast cancer was based on the seventh edition of the Union for International Cancer Control staging system [25, 26].

### Data source

This study utilized data from the National Clinical Database-Breast Cancer Registry (NCD-BCR), a nationwide web-based registry. It houses data of approximately 840,000 patients with breast cancer from more than 1,400 hospitals throughout Japan. This database contains detailed information on the patients’ demographics, clinicopathologic characteristics, treatment, and survival. The data were collected from hospitals across Japan, which covered > 90% of annual breast cancer cases in 2018 [27].

### Outcome measures

The primary outcome was to evaluate overall survival (OS) after ILRR diagnosis in relation to the PR status of the ILRR tumor. The secondary outcomes were to evaluate breast cancer-specific survival (BCSS) after ILRR diagnosis in relation to the PR status of the ILRR tumor and determine the prognostic factors associated with poor OS and BCSS after ILRR diagnosis. OS after ILRR was defined as the time from the date of the first ILRR event to the date of all-cause mortality. BCSS was defined as the time from the date of the first ILRR event to the date of death from breast cancer. Disease-free interval (DFI) was defined as the time from the initial surgery to the first detection of an ILRR event. Local recurrence was defined as the presence of a tumor in the ipsilateral breast after initial breast-conserving surgery (BCS) or the presence of a tumor in the chest wall (CW)/skin after initial mastectomy. Regional recurrence was defined as the presence of tumors in the regional node (RN), such as the internal mammary, supraclavicular, infraclavicular, or ipsilateral axillary nodes. The NCD database has registered locoregional recurrence sites in three categories: ipsilateral breast tumor recurrence (IBTR), axillary lymph node (ALN), and CW/skin/RN. For consistency, recurrence sites in this study were also classified into these three categories. In general, surgery for local recurrent tumors was defined as salvage mastectomy for IBTR and resection for CW/skin recurrence. The resection for axillary lymph node recurrence involved complete level I and II axillary lymph node dissection (ALND) after a prior sentinel node biopsy (SLNB) or resection of the recurrent tumor after a prior complete ALND. However, the NCD database includes only information on whether surgery for ILRR was performed. Detailed surgical procedures and whether the resection was curative were not recorded. The patients were divided into three groups. Group 1 included patients with a positive PR status for both primary and recurrent tumors (pPR+/rPR+). Group 2 included patients with PR+ primary and PR- recurrent tumors (pPR+/rPR-). Group 3 included patients with a negative PR status for both primary and recurrent tumors (pPR-/rPR-).

### Statistical analysis

The clinicopathological characteristics and treatment data were tabulated separately for the three groups. OS and BCSS after ILRR among the three groups were estimated using the Kaplan–Meier method, and survival estimates were compared using the log-rank test. Patients were censored at their last registered follow-ups. In the BCSS analysis, death not related to breast cancer was also censored. Multivariable Cox proportional hazard regression analysis was used to evaluate the independent prognostic contributions of each variable on OS and BCSS after ILRR. All statistical analyses were conducted using SAS, version 9.4 (SAS Institute, North Carolina, USA). Statistical significance was set at *P* < 0.05.

## Results

### Patient characteristics

**Fig. 1:**
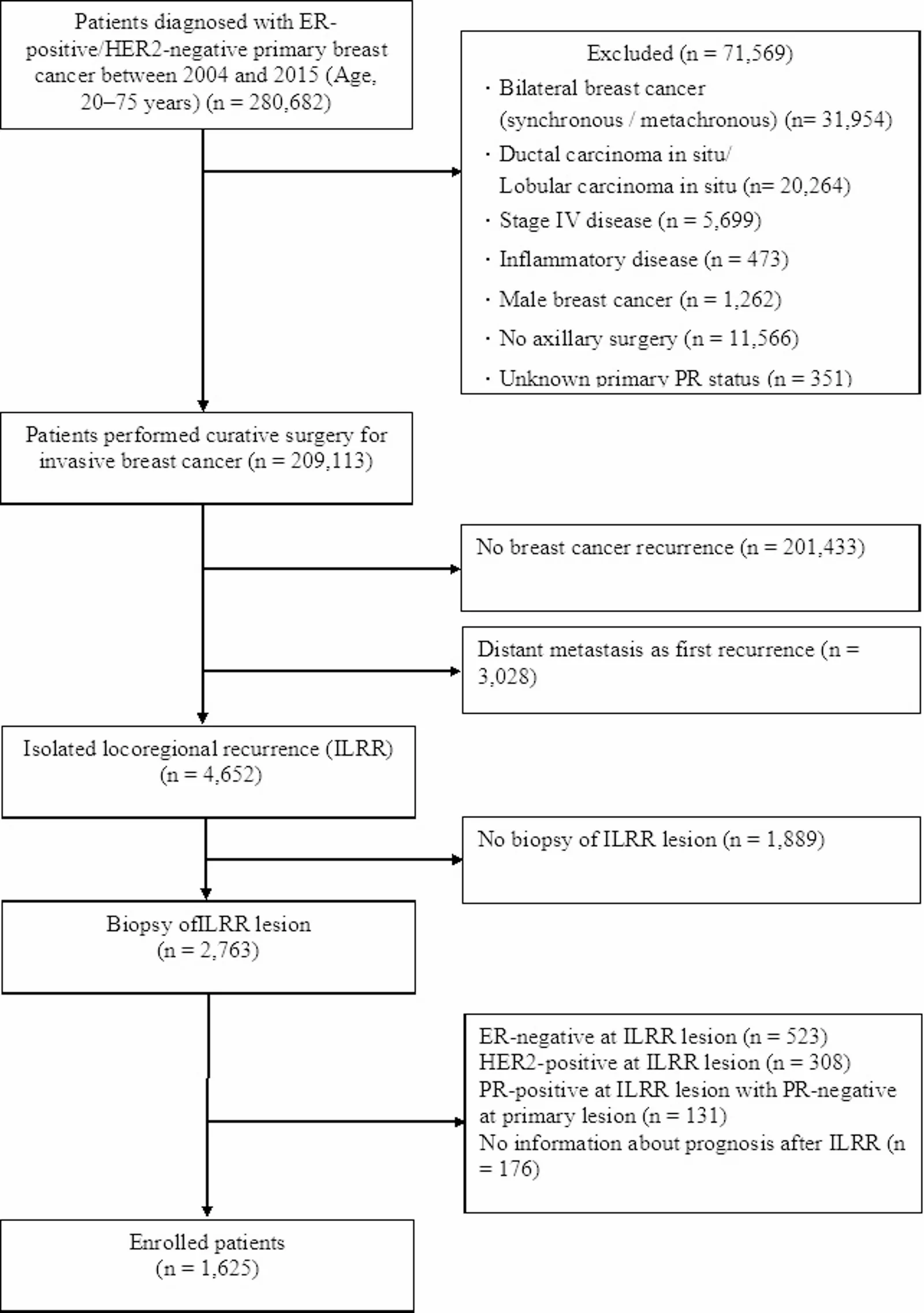
Flowchart illustrating the selection of patients included in this study. ER, estrogen receptor; PR, progesterone receptor; HER2, human epidermal growth factor receptor 2; ILRR, isolated locoregional recurrence

A total of 4,652 patients with ER+/HER2- ILRR tumors were identified in the database. Of these, 1,889 patients were excluded due to the absence of a biopsy of the ILRR lesion. After applying additional biological criteria, a further 1,138 patients were excluded, resulting in a final study cohort of 1,625 patients. The CONSORT diagram and patient characteristics are presented in Fig. 1; Table 1, respectively. The median follow-up time after ILRR diagnosis was 1,254 days (range: 1–1,825 days). During the follow-up period, 148 patients died: 129 died from breast cancer and the remaining 19 died from causes other than breast cancer. Among the 1,625 patients, 1,180 (72.6%) were in Group 1, 306 (18.8%) were in Group 2 and 139 (8.6%) were in Group 3. The distribution of recurrence sites was as follows: Group 1—IBTR 47%, ALN 28%, CW/skin/RN 35%; Group 2—IBTR 38%, ALN 38%, CW/skin/RN 42%; and Group 3—IBTR 34%, ALN 31%, CW/skin/RN 48%. No substantive differences were found in pathological tumor size, pathological nodal status of the primary breast cancer, type of breast and axillary surgery for primary breast cancer, adjuvant chemotherapy, adjuvant endocrine therapy, or radiotherapy between the three groups. Although not statistically significant, compared to patients in Group 1, patients in Group 2 tended to have a lower proportion of nuclear grade (NG)1 (12.1% vs. 7.8%), larger tumor size (38.3% vs. 48.4% ), higher incidence of lymph node metastasis (34.6% vs. 46.4%), and higher rates of ALND (42.5% vs. 55.6%), neoadjuvant chemotherapy (17.7% vs. 24.8%), and adjuvant chemotherapy (25.1% vs. 32.7%). Regarding DFI, although not statistically significant, compared to patients in Group 1 (12.2%), a higher proportion of patients in Group 2 (17.6%) had < 24 months, and this proportion was even greater in Group 3 (23.7%). Regarding the type of recurrence, IBTR was more frequent in Group 1 (46.9%), whereas the proportion of IBTR was lower in Group 2 (38.2%) and Group 3 (33.8%). The proportion of post-menopausal patients was higher in Group 3 than in Groups 1 and 2. Although the proportion of patients with ER-low expression (1–10%) in recurrent tumors was higher in Group 3 than in Groups 1 and 2, the proportions of patients with ER+ (≥ 10%) remained high at 93.8% and 88.5% in Groups 2 and 3, respectively. No substantive differences in ILRR treatment were observed between the three groups. Approximately 80%, 30%, 80%, and 30% of patients underwent surgery, chemotherapy, endocrine therapy, and adjuvant radiotherapy, respectively.

**Table 1:** Patient characteristics stratified by PR status

Characteristics	Category	pPR+/rPR+		pPR+/rPR-		pPR-/rPR-	
		(*n* = 1180)		(*n* = 306)		(*n* = 139)	
		n	(%)	N	(%)	N	(%)
Age (y) (at primary)	< 35	64	(5.4)	14	(4.6)	2	(1.4)
	35–64	916	(77.6)	227	(74.2)	95	(68.4)
	≥ 65	200	(17)	65	(21.2)	42	(30.2)
Menopausal status	Pre	659	(55.9)	129	(42.2)	30	(21.6)
	Post	493	(41.8)	167	(54.6)	106	(76.3)
	Unknown	28	(2.4)	10	(3.3)	3	(2.2)
Histological type	IDC_NST	1063	(90.1)	260	(85)	108	(77.7)
	Others	117	(9.9)	46	(15)	31	(22.3)
NG* (at primary)	1	150	(12.7)	24	(7.8)	12	(8.6)
	2	152	(12.9)	37	(12.1)	23	(16.6)
	3	74	(6.3)	28	(9.2)	13	(9.4)
	Unknown	793	(67.2)	214	(69.9)	87	(62.6)
	Not assessed	11	(0.9)	3	(1)	4	(2.9)
pT size (cm) (at primary)	≤ 2	694	(58.8)	148	(48.4)	81	(58.3)
	> 2	452	(38.3)	148	(48.4)	53	(38.1)
	Unknown	34	(2.9)	10	(3.3)	5	(3.6)
pN status (at primary)	Negative	772	(65.4)	164	(53.6)	96	(69.1)
	Positive	408	(34.6)	142	(46.4)	43	(30.9)
Type of breast surgery (at primary)	BCS	951	(80.6)	237	(77.5)	101	(72.7)
	MX	229	(19.4)	69	(22.5)	38	(27.3)
Type of axillary surgery (at primary)	SLNB	679	(57.5)	136	(44.4)	68	(48.9)
	ALND	501	(42.5)	170	(55.6)	71	(51.1)
Neoadjuvant therapy	Yes	209	(17.7)	76	(24.8)	37	(26.6)
	No	971	(82.3)	229	(74.8)	102	(73.4)
	Unknown	0	(0)	1	(0.3)	0	(0)
	CT	149	(12.6)	61	(19.9)	28	(20.1)
	ET	75	(6.4)	20	(6.5)	9	(6.5)
Adjuvant chemotherapy	Yes	296	(25.1)	100	(32.7)	29	(20.9)
	No	873	(74)	202	(66)	108	(77.7)
	Missing	11	(0.9)	4	(1.3)	2	(1.4)
Adjuvant endocrine therapy	Yes	980	(83.1)	270	(88.2)	114	(82)
	No	190	(16.1)	32	(10.5)	23	(16.6)
	Missing	10	(0.9)	4	(1.3)	2	(1.4)
Adjuvant radiotherapy	Yes	412	(34.9)	119	(38.9)	44	(31.7)
	No	754	(63.9)	182	(59.5)	93	(66.9)
	Missing	14	(1.2)	5	(1.6)	2	(1.4)
DFI (months)	< 24	144	(12.2)	54	(17.6)	33	(23.7)
	≥ 24	1036	(87.8)	252	(82.4)	106	(76.3)
Type of recurrence	IBTR	553	(46.9)	117	(38.2)	47	(33.8)
	Axillary lymph node	328	(27.8)	117	(38.2)	43	(30.9)
	Chest wall/skin/regional node	411	(34.8)	128	(41.8)	67	(48.2)
ER status of ILRR tumor (%)	1–10	24	(2)	19	(6.2)	16	(11.5)
	≥ 10	1156	(98)	287	(93.8)	123	(88.5)
Surgery for ILRR	Yes	1008	(85.4)	239	(78.1)	110	(79.1)
	No	172	(14.6)	67	(21.9)	29	(20.9)
CT for ILRR	Yes	242	(20.5)	99	(32.4)	41	(29.5)
	No	938	(79.5)	207	(67.7)	98	(70.5)
ET for ILRR	Yes	954	(80.9)	246	(80.4)	106	(76.3)
	No	226	(19.2)	60	(19.6)	33	(23.7)
Radiotherapy for ILRR	Yes	332	(28.1)	80	(26.1)	43	(30.9)
	No	848	(71.9)	226	(73.9)	96	(69.1)

*ILRR* isolated locoregional recurrence, *pT* pathological tumor, *IDC_NST* invasive ductal carcinoma of no special type, *NG* nuclear grade, *pN* pathological nodal, *pPR* primary tumor progesterone receptor, *rPR* recurrent tumor progesterone receptor, *HER2* human epidermal growth factor receptor 2, *BCS* breast-conserving surgery, *MX* mastectomy, *DFI* disease-free interval, *SLNB* sentinel lymph node biopsy, *ALND* axillary lymph node dissection, *CT* chemotherapy, *ET* endocrine therapy

*Nuclear grade has been registered since 2013

### OS after ILRR diagnosis based on the three groups

**Fig. 2:**
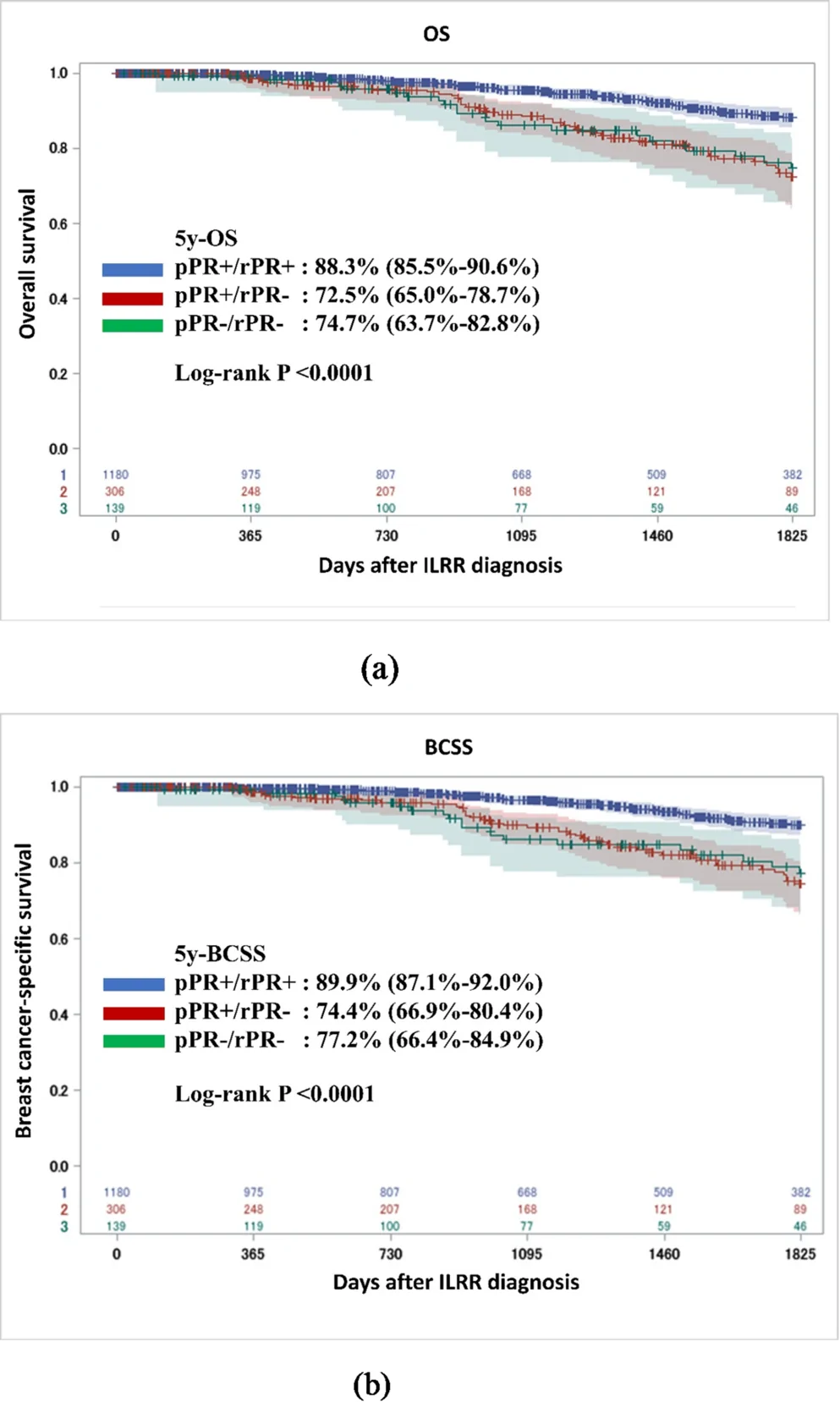
**a** Overall survival among patients with ER+/HER2- breast cancer, stratified by PR status. ER, estrogen receptor; PR, progesterone receptor; HER2, human epidermal growth factor receptor 2; ILRR, isolated locoregional recurrence; OS overall survival; p- primary; r- recurrent, **b** Breast cancer-specific survival among patients with ER+/HER2- breast cancer, stratified by PR status. ER estrogen receptor; PR progesterone receptor; HER2 human epidermal growth factor receptor 2; ILRR isolated locoregional recurrence; BCSS breast cancer-specific survival; p- primary; r- recurrent

Patients in Groups 2 and 3 had significantly worse 5-year OS after ILRR diagnosis than those in Group 1 (72.5% vs. 88.3%, *P* < 0.0001; 74.7% vs. 88.3%, *P* < 0.0001, respectively) (Fig. 2(a)). Multivariable analysis revealed that lymph node metastasis at primary breast cancer diagnosis (hazard ration [HR] 2.46; 95% confidence interval [CI] 1.69–3.60; *P* < 0.01), DFI shorter than 24 months (HR 1.99; 95% CI 1.38–2.88; *P* < 0.01), pPR+/rPR- tumor (HR 1.72; 95% CI 1.17–2.53; *P* < 0.01), pPR-/rPR- tumor (HR 1.83; 95% CI 1.08–3.09; *P* = 0.02), ER-low expression at ILRR tumors (HR 2.15; 95% CI 1.15–4.02; *P* = 0.02), and chemotherapy administered for ILRR (HR 1.65; 95% CI 1.14–2.39; *P* < 0.01) were associated with worse OS after ILRR diagnosis. Surgery for ILRR was associated with favorable OS after ILRR diagnosis (HR 0.35; 95% 0.24–0.51; *P* < 0.01). A pathological tumor size > 2 cm at primary breast cancer diagnosis tended to be a poor prognostic factor (HR 1.38; 95% CI 0.94–2.02; *P* = 0.10), and endocrine therapy administered for ILRR tended to be a favorable prognostic factor (HR 0.72; 95% CI 0.48–1.07; *P* = 0.11) (Table 2). In addition to direct comparison between Group 1 and Group 2, to evaluate the impact of primary PR status on OS after ILRR diagnosis, additional analysis was performed between patients with pPR+ (i.e., Group 1 plus Group 2) and those with pPR- (i.e., Group 3). Patient characteristics for those with pPR + and pPR- status are summarized in Supplementary Table 1. Compared with patients with pPR+, those with pPR- were more likely to be aged 65 years or older and to be postmenopausal. No substantive differences in treatment for either primary breast cancer or ILRR were observed between the two groups. Patients with pPR- had significantly worse 5-year OS after ILRR diagnosis than those with pPR+ (74.7% vs. 84.9%, *P* = 0.0076) (Supplementary Fig. 1(a).

**Table 2:** Multivariable analysis of OS after ILRR diagnosis

Characteristics	Category	HR	95% CI	*P*-value
Age (y) (at primary)	< 35	1.27	0.61–2.66	0.52
	35–64	1		
	≥ 65	1.32	0.88–1.98	0.18
Type of breast surgery (at primary)	MX	1		
	BCS	1.34	0.86–2.09	0.2
pT size (cm) (at primary)	≤ 2	1		
	> 2	1.38	0.94–2.02	0.1
pN status (at primary)	Negative	1		
	Positive	2.46	1.69–3.60	< 0.01
DFI (months)	≥ 24	1		
	< 24	1.99	1.38–2.88	< 0.01
Type of recurrence	None	1		
	IBTR	0.97	0.56–1.69	0.91
Type of recurrence	None	1		
	Axillary lymph node	1.35	0.85–2.12	0.2
Type of recurrence	None	1		
	Chest wall/skin/regional node	1.23	0.75–2.00	0.42
PR status	pPR+/rPR+	1		
	pPR+/rPR-	1.72	1.17–2.53	< 0.01
	pPR-/rPR-	1.83	1.08–3.09	0.02
ER status of ILRR tumors (%)	1–10	2.15	1.15–4.02	0.02
	≥ 10	1		
Surgery for ILRR	Yes	0.35	0.24–0.51	< 0.01
	No	1		
CT for ILRR	Yes	1.65	1.14–2.39	< 0.01
	No	1		
ET for ILRR	Yes	0.72	0.48–1.07	0.11
	No	1		
Radiotherapy for ILRR	Yes	1.11	0.76–1.63	0.59
	No	1		

OS overall survival, ILRR isolated locoregional recurrence, HR hazard ratio, CI confidential interval, pT pathological tumor, IDC_NST invasive ductal carcinoma of no special type, pN pathological nodal, pPR primary tumor progesterone receptor, rPR recurrent tumor progesterone receptor, HER2 human epidermal growth factor receptor 2, BCS breast-conserving surgery, MX mastectomy, DFI disease-free interval, CT chemotherapy, ET endocrine therapy

### BCSS after ILRR diagnosis based on the three groups

Patients in Groups 2 and 3 had significantly worse 5-year BCSS after ILRR diagnosis than those in Group 1 (74.4% vs. 89.9%, *P* < 0.0001; 77.2% vs. 89.9%, *P* < 0.0001, respectively) (Fig. 2(b)). Multivariable analysis revealed that lymph node metastasis at primary breast cancer diagnosis (HR 2.36; 95% CI 1.56–3.55; *P* < 0.01), DFI shorter than 24 months (HR 2.28; 95% CI 1.55–3.37; *P* < 0.01), pPR+/rPR- tumor (HR 1.90; 95% CI 1.26–2.88; *P* < 0.01), pPR-/rPR- tumor (HR 2.02; 95% CI 1.15–3.55; *P* = 0.02), ER-low expression at ILRR tumors (HR 2.12; 95% CI 1.10–4.10; *P* = 0.03), and chemotherapy administered for ILRR (HR 1.90; 95% CI 1.28–2.82; *P* < 0.01) were associated with worse BCSS after ILRR diagnosis. Surgery for ILRR was associated with favorable BCSS after ILRR diagnosis (HR 0.36; 95% 0.24–0.54; *P* < 0.01) (Table 3).

In addition to a direct comparison between Group 1 and Group 2, to evaluate the impact of primary PR status on BCSS after ILRR diagnosis, additional analysis was performed between patients with pPR+ (i.e., Group 1 plus Group 2) and pPR- (i.e., Group 3). Patients with pPR- had significantly worse 5-year BCSS after ILRR diagnosis than those with pPR+ (77.2% vs. 86.5%, *P* = 0.0059) (Supplementary Fig. 1(b)).

**Table 3:** Multivariable analysis of BCSS after ILRR diagnosis

Characteristics	Category	HR	95% CI	*P*-value
Age (y) (at primary)	< 35	1.18	0.54–2.60	0.68
	35–64	1		
	≥ 65	1.08	0.68–1.71	0.75
Type of breast surgery (at primary)	MX	1		
	BCS	1.45	0.90–2.34	0.13
pT size (cm) (at primary)	≤ 2	1		
	> 2	1.39	0.92–2.10	0.12
pN status (at primary)	Negative	1		
	Positive	2.36	1.56–3.55	< 0.01
DFI (months)	≥ 24	1		
	< 24	2.28	1.55–3.37	< 0.01
Type of recurrence	None	1		
	IBTR	0.85	0.47–1.55	0.6
Type of recurrence	None	1		
	Axillary lymph node	1.44	0.89–2.34	0.13
Type of recurrence	None	1		
	Chest wall/skin/regional node	1.3	0.78–2.18	0.32
PR status	pPR+/rPR+	1		
	pPR+/rPR-	1.9	1.26–2.88	< 0.01
	pPR-/rPR-	2.02	1.15–3.55	0.02
ER status of ILRR tumors (%)	1–10	2.12	1.10–4.10	0.03
	≥ 10	1		
Surgery for ILRR	Yes	0.36	0.24–0.54	< 0.01
	No	1		
Chemotherapy for ILRR	Yes	1.9	1.28–2.82	< 0.01
	No	1		
Endocrine therapy for ILRR	Yes	0.76	0.49–1.17	0.21
	No	1		
Radiotherapy for ILRR	Yes	1.18	0.79–1.77	0.42
	No	1		

BCSS breast cancer specific survival, ILRR isolated locoregional recurrence, HR hazard ratio, CI confidential interval, pT pathological tumor, IDC_NST invasive ductal carcinoma of no special type, pN pathological nodal, pPR, primary tumor progesterone receptor, rPR recurrent tumor progesterone receptor, HER2 human epidermal growth factor receptor 2, BCS breast-conserving surgery, MX mastectomy, DFI disease-free interval, CT chemotherapy, ET endocrine therapy

## Discussion

This study investigated the prognostic impact of PR status in patients with ER+/HER2- ILRR tumors using data from the Japanese National Clinical Database. The study included 1,625 patients with ER+/HER2- ILRR tumors, representing, to our knowledge, the largest number of patients with ILRR analyzed to date. Our findings revealed that compared to patients with PR + in both the primary and ILRR tumors (i.e., Group 1), patients with PR- in both the primary and ILRR tumors (i.e., Group 3) had significantly worse OS and BCSS after ILRR. Similarly, patients who were PR + in the primary tumor but became PR- in the ILRR tumors (i.e., Group 2) also had worse prognosis.

Regarding the prognostic impact of primary PR status, many studies have explored the prognosis of ER+ primary breast cancer stratified by the PR status of the primary tumor; these studies have indicated that ER+/PR-/HER2- primary breast cancer has distinct clinicopathological features compared with ER+/PR+/HER2- primary breast cancer. Additionally, several studies have shown that, compared with patients with ER+/PR+ tumors, those with ER+/PR- tumors were more likely to be older and to have aggressive features, such as larger tumors, high-grade tumors, lymphovascular invasion, nodal metastasis, and higher mean multigene assay scores [28–31]. Other studies have indicated that patients with ER+/PR- tumors have a worse prognosis than those with ER+/PR+ tumors [32–34]. In line with previous studies, patients in our study with pPR- were more likely to be older, postmenopausal, and to have higher-grade tumors compared to patients with pPR+. Although the proportions of patients with nodal metastasis, tumor size > 2 cm, and receipt of neoadjuvant and adjuvant chemotherapy were similar between the pPR + and pPR- groups, the pPR− group had higher proportions of patients with a DFI < 24 months and with recurrence patterns other than IBTR, suggesting more aggressive features of pPR- tumors. However, reports specifically examining the prognostic impact of PR status in ILRR tumors are limited [12, 13], and since none of these reports address the effect of primary tumor PR status, direct comparison with our findings is not possible.

Regarding the effect of PR loss, a direct comparison between Group 1 and Group 2 demonstrated that patients in Group 2 had significantly worse 5-year OS and BCSS after ILRR than those in Group 1. A large meta-analysis of steroid hormone receptor discordance in primary and recurrent breast cancers estimated the frequency of secondary PR loss at 46% in patients with local and distant metastases [35]. Another study reported that down-regulation of ERα and PR can be induced by endocrine therapy [36]. The loss of ERα and/or PR in relapsing tumors or after primary systemic treatment probably indicates the selection of hormone receptor-negative cells from a heterogeneous tumor cell population [37]. Additionally, circulating tumor cells (CTCs) have been shown to frequently exhibit discordant profiles with primary tumors. PR-CTCs are reported to be present in 68–87% of patients with PR+ primary tumors, and this pool may contribute to ER+/PR- recurrences [38]. Considering that our results showed a slightly higher proportion of adjuvant endocrine therapy in Group 2 patients (88%) than that in Group 1 patients (83%), PR loss in Group 2 patients may have been more influenced by adjuvant endocrine therapy.

Regarding the efficacy of endocrine therapy, ER+/PR-/HER2- tumors show lower endocrine responsiveness than ER+/PR+/HER2- tumors [28, 34, 39, 40]. In terms of genomic features, Liu et al. reported that 97% of ER+/PR+/HER2- tumors were classified in the luminal-like subgroup defined by PAM50. In contrast, only 75% of ER+/PR-/HER2- tumors were classified as luminal-like, while approximately 20% were classified into the non-luminal-like subgroup defined by PAM50, with low endocrine sensitivity scores [41]. A higher rate of TP53 mutations has been reported in ER+/PR- breast cancers than in ER+/PR+ breast cancers. Some clinical trials indicate that TP53-mutated luminal breast cancers are resistant to endocrine therapy with TAM and aromatase inhibitors [42, 43]. Regarding chemotherapy, patients with ER+/PR- tumors are more sensitive to chemotherapy than those with ER+/PR+ tumors. Additionally, PR-negativity is an independent predictive factor for pathological complete response after neoadjuvant chemotherapy [33, 44]. Therefore, since PR-negativity in ER+ primary breast cancer is associated with tumor aggressiveness and resistance to endocrine therapy, PR-negativity in ER+ ILRR is also likely associated with tumor aggressiveness and resistance to endocrine therapy. The CALOR trial showed no benefit of chemotherapy for patients with ER+ ILRR, but this trial did not examine patients by PR status. Considering our results, the treatment of ER+/PR+ ILRR and ER+/PR- ILRR should be considered different subtypes requiring different treatment strategies. ER+/PR- ILRR is presumed to be less sensitive to endocrine therapy and more sensitive to chemotherapy compared with ER+/PR+ breast cancer. In the present study, chemotherapy for ILRR was a poor prognostic factor in the multivariable analysis, possibly because chemotherapy was administered to patients who were considered more likely to have a poorer prognosis. Since this was a retrospective analysis, confounding factors may have been present. Although the prognostic benefit of chemotherapy may be greater in patients with PR- ILRR than in those with PR+ ILRR, the present study could not adequately assess this because only approximately 20% of patients with PR+ ILRR and 30% of those with PR- ILRR received chemotherapy. This requires further evaluation in future prospective studies.

ER-low positive/HER2- breast cancer is similar to ER-/HER2- breast cancer in terms of molecular landscape, clinicopathological characteristics, prognosis, and response to therapy [45, 46]. In our study, ER-low positivity of ILRR tumors was shown to be an independent poor prognostic factor. In addition, PR-negativity of ILRR tumors was also shown to be an independent poor prognostic factor, which is meaningful because it indicates that even if the ER expression level of the ILRR tumor is more than 10%, if the tumor is PR-, the prognosis is poor. To the best of our knowledge, this is the first study to demonstrate that PR-negativity of ER+/HER2- ILRR is a poor prognostic factor, independent of the PR status of the primary breast cancer and regardless of the ER expression level (1–10%, ≥ 10%) of the ILRR tumor, in a large cohort.

We excluded patients with pPR−/rPR+ from the present study. Previous clinicopathological classification frameworks have used tumor location, histologic subtype, and hormone receptor/HER2 profiles to distinguish TR from NP [14, 15, 17]. In this context, hormone receptor gain generally reflects a more differentiated phenotype and may indicate a biologically distinct tumor rather than clonal evolution of the original disease [18–20]. Because such cases are more compatible with NP than with recurrence of the original ER+/HER2 − tumor, their inclusion could have introduced misclassification bias in evaluating the prognostic impact of PR status.

In the additional descriptive analysis of this subgroup (*n* = 131), the 5-year OS and BCSS after ILRR were 86.8% and 88.6%, respectively, which were comparable to those of the pPR+/rPR+ group and more favorable than those of the pPR−/rPR− group. Nearly half of the ILRR events in this subgroup occurred within the conserved breast, a setting known to include both TR and NP. Although axillary, chest wall, and regional nodal recurrences are generally considered TR, the registry does not distinguish isolated axillary recurrences from combined breast-and-axillary events. Therefore, some cases categorized as axillary recurrences may have arisen from NP in the conserved breast with nodal involvement. This possibility, together with the favorable survival profile, further supports the interpretation that the pPR−/rPR+ subgroup represents a biologically heterogeneous mixture of TR and NP.

For these reasons, pPR−/rPR+ cases were excluded from the primary analyses; however, their baseline characteristics and crude outcomes are presented in Supplementary Tables 2 and Supplementary Fig. 2 for transparency.

Regarding adjuvant radiotherapy, considering that more than 80% of patients underwent breast-conserving surgery for primary breast cancer, the proportion of patients who received adjuvant radiotherapy was relatively small (34.9%). Possible reasons for this could be that radiotherapy registration was not performed perioperatively, or that radiotherapy registration was not performed in patients who received radiotherapy at other hospitals.

Distant metastasis-free survival and OS among patients with breast cancer who develop ILRR have been reported to be associated with age at primary breast cancer, type of surgery performed for primary breast cancer, tumor size, nodal metastasis, hormone receptor status of the primary tumor, DFI, and ILRR site [46–50]. Additionally, a single-center retrospective study also reported that ER+/PR-/HER2- ILRR, lack of resection of the ILRR tumor, and chemotherapy for the primary tumor were also associated with poor distant metastasis-free survival [13]. Consistent with previous reports, nodal metastasis, DFI, and surgery for ILRR were associated with prognosis in the present study. Regarding primary tumor size, a pathological tumor size greater than 2 cm at primary breast cancer tended to be a poor prognostic factor in the present study. In contrast, in a previous report, CW and RN recurrence were cited as poor prognostic factors with respect to the site of recurrence; however, no association was found in the present study. This may be attributable to our inability to distinguish among CW, skin recurrence, and RN recurrences due to the limitations in the classification system of our registry, which prevented us from analyzing the results in a manner consistent with previous reports. The inability to distinguish between multiple- and single-site recurrence may also have influenced our analysis. In the present study, younger age at initial onset was not a poor prognostic factor, but the fact that only 5% of patients (*n* = 80) were younger than 35 years of age may have influenced our analysis.

Several limitations of this study should be acknowledged. First, the study had a retrospective design. Second, the database did not include information on distant metastasis after ILRR diagnosis. Third, baseline characteristics differed between the three groups, and selection bias may have influenced the treatment patterns. Fourth, patients who did not undergo biopsy for ILRR tumors were excluded (*n* = 1,889, Fig. 1) because the inclusion criteria required assessment of the ILRR tumor receptor status. The reasons for not performing a biopsy may include recurrence at sites where both core needle biopsy and fine needle aspiration cytology were difficult to perform, or only fine needle aspiration cytology could be performed because core needle biopsy or resection of the recurrence site was difficult. We could not evaluate the impact of excluding these cases from the present analysis. Fifth, we could not confirm whether surgery for ILRR presented curative resection since no detailed information was registered in the NCD database. Sixth, the follow-up period after ILRR diagnosis was relatively short for assessing the survival outcomes for patients with ER+/HER2- ILRR. However, to our knowledge, this is the largest cohort study to investigate the prognostic impact of PR status in ER+/HER2- ILRR among patients with ER+/HER2- primary breast cancer.

In conclusion, the results from this nationwide database study demonstrated that compared to patients who were PR + in both the primary and ILRR tumors, patients who were PR- in both the primary and ILRR tumors had a worse prognosis. Similarly, patients who were PR + in the primary tumor but became PR- in the ILRR tumors also had a worse prognosis.

## Supplementary Information

Below is the link to the electronic supplementary material.Supplementary file1 (DOCX 30 KB)Supplementary file2 (DOCX 502 KB)

## Data Availability

The data that support the findings of this study are available from the Japanese Breast Cancer Society and the Committee of Breast Cancer Registry and Data Science, but restrictions apply. The data were used under license, and therefore are not publicly available. However, the data can be made available from the authors upon reasonable request as long as permission is obtained from the Japanese Breast Cancer Society, the Committee of Breast Cancer Registry and Data Science, and the National Clinical Database.
