# Supplementary material for: Prognostic impact of progesterone receptor status in patients with breast cancer and isolated locoregional recurrence
Source: Breast Cancer. 2026 Jun 21;33(5):1070–81. doi: 10.1007/s12282-026-01882-z (PMC13522001; doi:10.1007/s12282-026-01882-z)
Supplement: Supplementary file 1 [file 12282_2026_1882_MOESM1_ESM.docx]

**Supplementary Table 1. Patients characteristics stratified by primary PR status**

| Characteristics | Category | pPR+  (n=1486) | | pPR-  (n=139) | |
| --- | --- | --- | --- | --- | --- |
|  |  | n | (%) | N | (%) |
| Age (y)  (at primary) | < 35  35-64  ≥ 65 | 78  1143  265 | (5.2)  (76.9)  (17.8) | 2  95  42 | (1.4)  (68.4)  (30.2) |
| Menopausal status | Pre  Post  Unknown | 788  660  38 | (53.0)  (44.4)  (2.6) | 30  106  3 | (21.6))  (76.3)  (2.2) |
| Histological type | IDC_NST  Others | 1323  163 | (89.0)  (11.0) | 108  31 | (77.7)  (22.3) |
| NG*  (at primary) | 1  2  3  Unknown  Not assessed | 174  191  103  1004  14 | (11.2)  (12.1)  (6.7)  (69.1)  (0.9) | 12  23  13  87  4 | (8.6)  (16.6)  (8.6)  (63.3)  (2.9) |
| pT size (cm)  (at primary) | ≤ 2  > 2  Unknown | 842  600  44 | (56.7)  (40.4)  (3.0) | 81  53  5 | (58.3)  (38.1)  (3.6) |
| pN status  (at primary) | Negative  Positive | 936  550 | (63.0)  (37.0) | 96  43 | (69.1)  (30.9) |
| Type of breast surgery  (at primary) | BCS  MX | 1188  298 | (79.9)  (20.1) | 101  38 | (72.7)  (27.3) |
| Type of axillary surgery  (at primary) | SLNB  ALND | 815  671 | (54.8)  (45.2) | 68  71 | (48.9)  (51.1) |
| Neoadjuvant therapy | Yes  No  Unknown  CT  ET | 285  1200  1  210  95 | (19.2)  (80.8)  (0.1)  (14.1)  (6.4) | 37  102  0  28  9 | (26.6)  (73.4)  (0.0)  (20.1)  (6.5) |
| Adjuvant chemotherapy | Yes  No  Missing | 396  1075  15 | (26.6)  (72.3)  (1.0) | 29  108  2 | (20.9)  (77.7)  (1.4) |
| Adjuvant endocrine therapy | Yes  No  Missing | 1250  222  14 | (84.1)  (14.9)  (0.9) | 114  23  2 | (82.0)  (16.6)  (1.4) |
| Adjuvant radiotherapy | Yes  No  Missing | 531  936  19 | (35.7)  (63.0)  (1.3) | 44  93  2 | (31.7)  (66.9)  (1.4) |
| DFI (months) | < 24  ≥ 24 | 198  1288 | (13.3)  (86.7) | 33  106 | (23.7)  (76.3) |
| Type of recurrence | IBTR  Axillary lymph node  Chest wall/skin/regional node | 670  445  539 | (45.1)  (29.9)  (36.3) | 47  43  67 | (33.8)  (30.9)  (48.2) |
| ER status of ILRR tumor (%) | 1–10  ≥ 10 | 46  1443 | (3.1)  (97.1) | 16  123 | (11.5)  (88.5) |
| Surgery for ILRR | Yes  No | 1247  239 | (83.9)  (16.1) | 110  29 | (79.1)  (20.9) |
| CT for ILRR | Yes  No | 341  1145 | (22.9)  (77.1) | 41  98 | (29.5)  (70.5) |
| ET for ILRR | Yes  No | 1200  286 | (80.8)  (19.2) | 106  33 | (76.3)  (23.7) |
| Radiotherapy for ILRR | Yes  No | 421  1074 | (28.3)  (72.3) | 43  96 | (30.9)  (69.1) |

Abbreviations: ILRR, isolated locoregional recurrence; pT, pathological tumor; IDC_NST, invasive ductal carcinoma of no special type; NG, nuclear grade; pN, pathological nodal; pPR, primary tumor progesterone receptor; rPR, recurrent tumor progesterone receptor; HER2, human epidermal growth factor receptor 2; BCS, breast-conserving surgery; MX, mastectomy; DFI, disease-free interval; SLNB, sentinel lymph node biopsy; ALND, axillary lymph node dissection; CT, chemotherapy; ET, endocrine therapy

*Nuclear grade has been registered since 2013

**Supplementary Table 2. Baseline characteristics of patients with pPR−/rPR+ tumors**

| Characteristics | Category | pPR-/rPR+  (n=131) | |
| --- | --- | --- | --- |
|  |  | n | (%) |
| Age (y)  (at primary) | < 35  35-64  ≥ 65 | 78  1143  265 | (5.2)  (76.9)  (17.8) |
| Menopausal status | Pre  Post  Unknown | 788  660  38 | (53.0)  (44.4)  (2.6) |
| Histological type | IDC_NST  Others | 1323  163 | (89.0)  (11.0) |
| NG*  (at primary) | 1  2  3  Unknown  Not assessed | 174  191  103  1004  14 | (11.2)  (12.1)  (6.7)  (69.1)  (0.9) |
| pT size (cm)  (at primary) | ≤ 2  > 2  Unknown | 842  600  44 | (56.7)  (40.4)  (3.0) |
| pN status  (at primary) | Negative  Positive | 936  550 | (63.0)  (37.0) |
| Type of breast surgery  (at primary) | BCS  MX | 1188  298 | (79.9)  (20.1) |
| Type of axillary surgery  (at primary) | SLNB  ALND | 815  671 | (54.8)  (45.2) |
| Neoadjuvant therapy | Yes  No  Unknown  CT  ET | 285  1200  1  210  95 | (19.2)  (80.8)  (0.1)  (14.1)  (6.4) |
| Adjuvant chemotherapy | Yes  No  Missing | 396  1075  15 | (26.6)  (72.3)  (1.0) |
| Adjuvant endocrine therapy | Yes  No  Missing | 1250  222  14 | (84.1)  (14.9)  (0.9) |
| Adjuvant radiotherapy | Yes  No  Missing | 531  936  19 | (35.7)  (63.0)  (1.3) |
| DFI (months) | < 24  ≥ 24 | 198  1288 | (13.3)  (86.7) |
| Type of recurrence | IBTR  Axillary lymph node  Chest wall/skin/regional node | 670  445  539 | (45.1)  (29.9)  (36.3) |
| ER status of ILRR tumor (%) | 1–10  ≥ 10 | 46  1443 | (3.1)  (97.1) |
| Surgery for ILRR | Yes  No | 1247  239 | (83.9)  (16.1) |
| CT for ILRR | Yes  No | 341  1145 | (22.9)  (77.1) |
| ET for ILRR | Yes  No | 1200  286 | (80.8)  (19.2) |
| Radiotherapy for ILRR | Yes  No | 421  1074 | (28.3)  (72.3) |

Abbreviations: ILRR, isolated locoregional recurrence; pT, pathological tumor; IDC_NST, invasive ductal carcinoma of no special type; NG, nuclear grade; pN, pathological nodal; pPR, primary tumor progesterone receptor; rPR, recurrent tumor progesterone receptor; HER2, human epidermal growth factor receptor 2; BCS, breast-conserving surgery; MX, mastectomy; DFI, disease-free interval; SLNB, sentinel lymph node biopsy; ALND, axillary lymph node dissection; CT, chemotherapy; ET, endocrine therapy

*Nuclear grade has been registered since 2013
