# Supplementary material for: Prognostic impact of progesterone receptor status in patients with breast cancer and isolated locoregional recurrence
Source: Breast Cancer. 2026 Jun 21;33(5):1070–81. doi: 10.1007/s12282-026-01882-z (PMC13522001; doi:10.1007/s12282-026-01882-z)
Supplement: Supplementary file 2 [file 12282_2026_1882_MOESM2_ESM.docx]

Supplementary Figure 1(a)

Supplementary Figure 1(b)

Supplementary Figure 2(a)

Supplementary Figure 2(b)
